# A Sound Source Localisation Analytical Method for Monitoring the Abnormal Night Vocalisations of Poultry

**DOI:** 10.3390/s18092906

**Published:** 2018-09-01

**Authors:** Xiaodong Du, Fengdan Lao, Guanghui Teng

**Affiliations:** 1 College of Water Conservancy & Civil Engineering, China Agricultural University, Beijing 100083, China; duxiaodong@cau.edu.cn; 2 Key Lab of Agricultural Engineering in Structure and Environment, Ministry of Agriculture, Beijing 100083, China; 3 Network Center, China Agricultural University, Beijing 100083, China; laofd@cau.edu.cn

**Keywords:** sound analysis, sound source localization, Kinects, chicken, animal behaviour

## Abstract

Due to the increasing scale of farms, it is increasingly difficult for farmers to monitor their animals in an automated way. Because of this problem, we focused on a sound technique to monitor laying hens. Sound analysis has become an important tool for studying the behaviour, health and welfare of animals in recent years. A surveillance system using microphone arrays of Kinects was developed for automatically monitoring birds’ abnormal vocalisations during the night. Based on the principle of time-difference of arrival (TDOA) of sound source localisation (SSL) method, Kinect sensor direction estimations were very accurate. The system had an accuracy of 74.7% in laboratory tests and 73.6% in small poultry group tests for different area sound recognition. Additionally, flocks produced an average of 40 sounds per bird during feeding time in small group tests. It was found that, on average, each normal chicken produced more than 53 sounds during the daytime (noon to 6:00 p.m.) and less than one sound at night (11:00 p.m.–3:00 a.m.). This system can be used to detect anomalous poultry status at night by monitoring the number of vocalisations and area distributions, which provides a practical and feasible method for the study of animal behaviour and welfare.

## 1. Introduction

Information about animals can be transferred over long distances by sound. The acoustic monitoring of farm animals is non-invasive and may be used as an efficient management tool to improve animal health, welfare and the economic efficiency of farms [1,2,3,4]. In recent years, interest in the analysis of farm animal vocalisation has grown, and various attempts have been made to decode the relationship between animal sound and animal behaviour [1]. For instance, some approaches have examined the relationship between vocalisation and the health of pigs and dairy calves [2,3,5,6,7,8]. Others suggest that vocalisations could serve as indicators of poultry welfare [9,10,11]. Sound analysis systems can be used to predict feed intake by monitoring pecking sounds [12,13,14]. Sound source localisation systems can be used to detect pig respiratory disease [15]. Some researchers have focused on vocalisation analysis of small-scale poultry breeding groups to model intensive cultivation [16,17]. However, unlike monitoring large farm animals, real-time sound analysis or sound recognition in a large-scale henhouse remain a great challenge since many sounds are produced at the same time. The basis of the sound analysis method is the identification of particular poultry sounds. Rather than simply monitor the whole house, this study focused on real-time poultry sound source localisation for precision research and quantitative analysis. Certainly, automatically captured abnormal sounds, such as respiratory disease symptoms (grunts, snores, and coughs) or noises from machine malfunctions at night, can help find sick chickens and solve problems quickly.

Sound source localisation (SSL) is one of the most significant sound analysis methods in acoustic research. It has been applied to video conferencing, surveillance and advanced human–computer interaction analyses [18] and for localisation of birds based on their vocalisations [19]. Microphone array technology can be used to study any animal that makes distinctive sounds [20], presenting an important and transformative tool for behavioural biologists. The logic behind placing microphones in different places is to identify the origin and direction of the incoming sound. Arrays of simultaneously recording microphones provide a useful tool for passively monitoring animals’ abnormal sounds, as well as ascertaining sound location. As the microphones are placed in different positions, the sound arrives at each microphone at a different time. By analysing the differences in sound arrival time from the source, sensors can calculate the direction and approximate distance from which the sound is coming [21,22].

This study aims to develop a non-invasive method to monitor abnormal night vocalisations of birds by using Kinect’s SSL technology. The objectives are as follows: (i) description of a sound source localisation method and experimental platform; (ii) measurement of the accuracy of this system; and (iii) application of SSL method to small poultry group tests.

## 2. Materials and Methods

Experiments were divided into laboratory tests that developed an SSL algorithm and small poultry group tests that evaluated the algorithm. All experimental procedures were conducted in conformity with hy-line parent stock management guides for the care and use of laboratory animals. All efforts were made to improve animal welfare and ameliorate the animals’ suffering.

### 2.1. Animals and Housing

**Figure 1:**
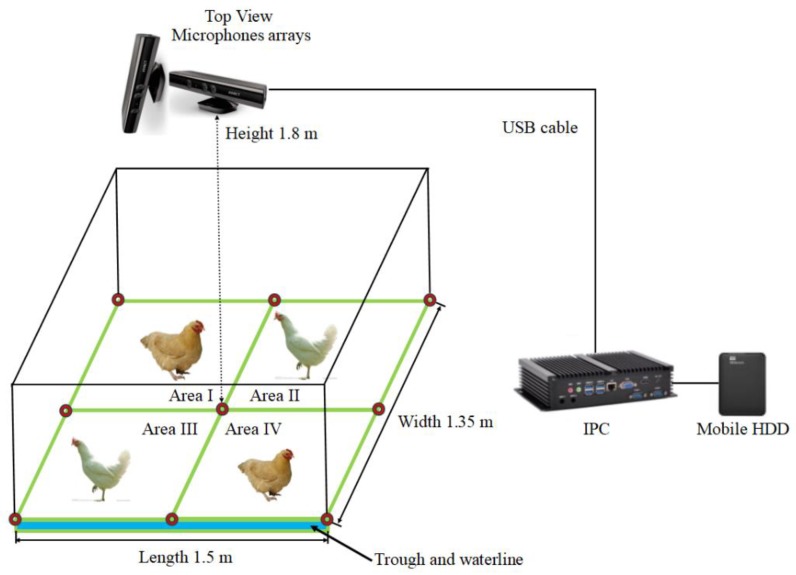
A schematic of the small group test platform. Note: Area I: laying area. Area II: activity area. Areas III and IV: feeding and drinking areas.

Small group tests were performed at the Shangzhuang Experimental Station of China Agricultural University, Beijing, China. Hy-line brown chickens (11 hens, 4 cocks) were raised on nets over a 36–38 week period, in a room 7.2 mL × 3.4 mW × 3.0 mH with a metal door and a surrounding wall made of colour steel plate. The floor-rearing area was 1.5 mL × 1.35 mW × 1.8 mH. The poultry flock was fed twice a day, once between noon and 1:00 p.m. and again between 5:00 and 6:00 p.m. Room temperature was kept at 15–18 °C, and 18 h light and 6 h dark were provided each day. The rearing area was divided into four sub areas: Area I was defined as a laying area; Area II was defined as an activity area; and Areas III and IV were both defined as feeding and drinking areas (Figure 1).

**Figure 2:**
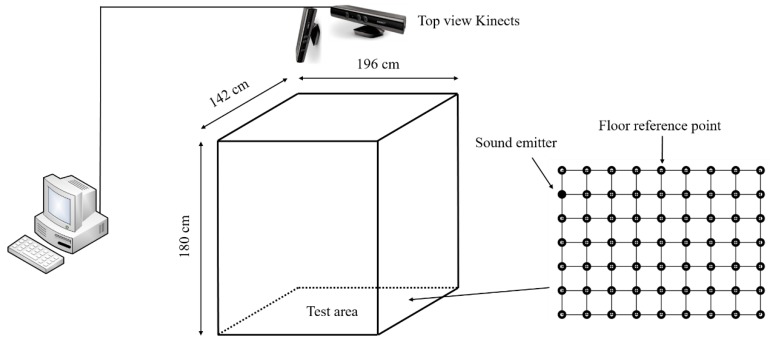
A schematic of the laboratory experiment platform.

The laboratory experiment platform was similar to the small group test platform, except for the sound source and test area size. The room (7.0 mL × 7.0 mW × 3.0 mH) had a metal door with a surrounding wall made of ferroconcrete. The size of the test area was 1.96 mL × 1.42 mW × 1.80 mH. In the experiment, a mobile phone (type: Meizu MX6, Helio X20 CPU, 4 GB RAM, 3 GB + 32 GB storage capacity, Meizu Telecom Equipment Co., Ltd., Guangdong, China ) was used as a sound emitter. It was placed in different locations as reference points (Figure 2). In this paper, we simplified animal vocalisation as a point of moving sound source without considering the animal’s direction. Pre-set floor reference points, regarded as truth values, were used to measure the accuracy of a single Kinect by comparing measured values with the known sound source angles, such as −28°, −22°, −15°, −8°, 0°, 8°, 15°, 22°, 28°. Then, errors of different angle measurements for a single Kinect were calculated to evaluate the accuracy of this system. The selected signal was a simulated laying hen call at a 44.1 kHz sampling rate (loop playback, duration 2.48 s, stereo sound channel, 16-bit bit depth, 1411 Kbps bit rate).

### 2.2. Experimental Installations

Two top-view Kinect cameras for Windows V1 (Microsoft Corp., Washington, WA, USA) were installed perpendicular to each other 1.8 m above the centre of the floor. Each Kinect was equipped with an RGB camera, a depth camera and a microphone array consisting of four different microphones placed in a linear order (three are placed on the right side and the other one is placed on the left side), sharing a common horizontal axis. From the left to the right, the distances between the microphones were 149 mm, 40 mm and 37 mm [23]. The microphone array could supply four channels of 32-bit audio at a 16 kHz sampling rate. Kinects continuously collected both digital images in JPEG format (640 × 480 pixel resolution, recorded at approximately 1 s intervals, used for manual verification) and multi-channel signal input (4-channel, 32-bit, 16,000 Hz, used for automatic sound source localisation measurements at 1 s intervals). Figure 1 shows that the microphone arrays were connected via two USB ports to one mini-industrial personal computer (IPC, Shenzhen Konghui Intellitech Co., Ltd., Shenzhen, China) for achieving coherence of the time series. It was also equipped with a 2 TB USB 3.0 mobile hard disk drive (HDD, Western Digital Corporation, LakeForest, IL, USA) to record data.

### 2.3. Sound Source Localisation Method

**Figure 3:**
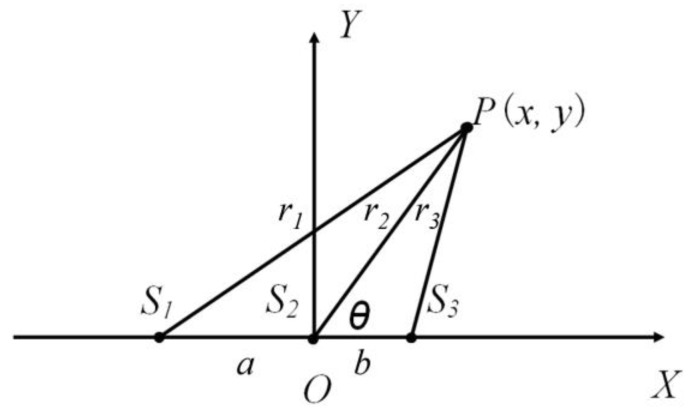
Linear localisation chart with any three microphones.

The SSL method is fundamentally based on the estimation of a sound source angle, a beam angle, and response to changes [23]. We combined Kinect Software Development Kits (SDK) such as NoiseSuppression, EchoCancellationMode and algorithms with LabVIEW sound and vibration modules to realise the online localisation function in our monitoring system. These Kinect SDK were called for sound signal preprocessing and the LabVIEW module was used to obtain the time delay. Because the microphones were placed in different positions, the sound arrived at different microphones at different times. As a result, the Kinect sensor determined the direction from which the sound came. Although Kinect position estimations are unsatisfactory, its direction estimations are very accurate based on the principle of time-difference of arrival (TDOA) and the steered response power using the PHAse Transform (SRP-PHAT) localization algorithm [24]. The reference algorithm and Kinect beamforming algorithm both need a large amount of calculation and are not suitable for real-time signal processing, while the cross correlation (CC) algorithm can realise the LabVIEW real-time localisation function with a small amount of calculation. Kinect signals were localised by extracting the time-difference of signal input in different microphones at 1 s intervals. 1 s was set as the SSL interval time because the movement of chickens was limited during that period. All four microphones shared a common horizontal axis, so a linear localisation could be detected with any three microphones (Figure 3). Though the Kinect device has 4 microphones, only three were utilised for SSL.

In Figure 3, it is supposed that the sound source and any three microphones are at point *P* (*x*, *y*), *S*_1_ (−*a*, 0), *S*_2_ (0, 0) and *S*_3_ (*b*, 0), respectively. Point *P* (*x*, *y*) can be represented by the angle θ (the included angle between line *S*_2_*S*_3_ and line *PS*_2_, “°”) and the distance between *P* and *S*_2_ (line segment PO, $r_{2}$), which can be calculated through a geometrical relationship:

(1) $$r_{1}=\sqrt{{\left(x+a\right)}^{2}+y^{2},}$$

(2) $$r_{2}=\sqrt{x^{2}+y^{2},}$$

(3) $$r_{3}=\sqrt{{\left(x-b\right)}^{2}+y^{2}},$$

where *r_i_* stands for the distance between the sound source point *P* and every microphone point *S_i_* (*i* = 1, 2, 3), m; *a* stands for the distance between point *S*_1_ and point *S*_2_, m; and *b* stands for the distance between points *S*_2_ and *S*_3_, m.

Assuming that sound velocity *c* = 340 m·s^−1^, *t*_12_ is the arriving time difference between *S*_1_ and *S*_2_, *s*, and *t*_23_ is the arriving time difference between *S*_2_ and *S*_3_, *s*. Time delay is automatically computed by Kinect adaptive methods embedded in the LabVIEW software and can be found from the triangle cosine theorem [25]:

(4) $${r_{1}}^{2}-r_{2}{}^{2}-2a\cdot r_{2}cos\theta =a^{2},$$

(5) $${r_{3}}^{2}-r_{2}{}^{2}+2b\cdot r_{2}cos\theta =b^{2},$$

(6) $$r_{1}-r_{2}=c\cdot t_{12,}$$

(7) $$r_{2}-r_{3}=c\cdot t_{23.}$$

Solve the equation using Equations (4)–(7) for $r_{2}$ and cos θ [26,27]:

(8) $$r_{2}=\frac{2b\cdot \left(c^{2}\cdot t_{12\;}{}^{2}-a^{2}\right)+2a\cdot \left(c^{2}\cdot t_{23}{}^{2}-b^{2}\right)}{4c\cdot \left(a\cdot t_{23}-b\cdot t_{12}\right)},$$

(9) $$\cos\theta =\frac{2t_{12}\cdot \left(b^{2}\cdot c-c^{3}\cdot t_{23}{}^{2}\right)+2t_{23}\cdot \left(a^{2}\cdot c-c^{3}\cdot t_{12}{}^{2}\right)}{2a\cdot \left(b^{2}-c^{2}\cdot t_{23}{}^{2}\right)+2b\cdot \left(a^{2}-c^{2}\cdot t_{12}{}^{2}\right)}.$$

**Figure 4:**
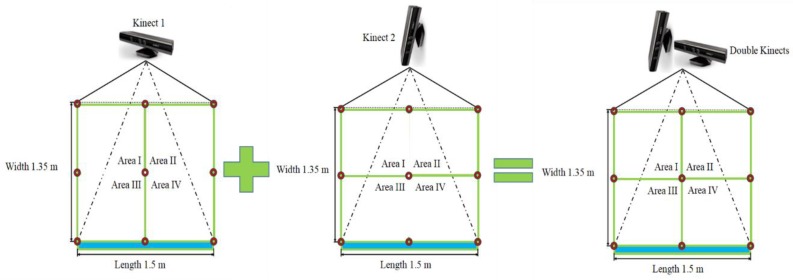
A schematic of Kinect quadrant estimations.

**Figure 5:**
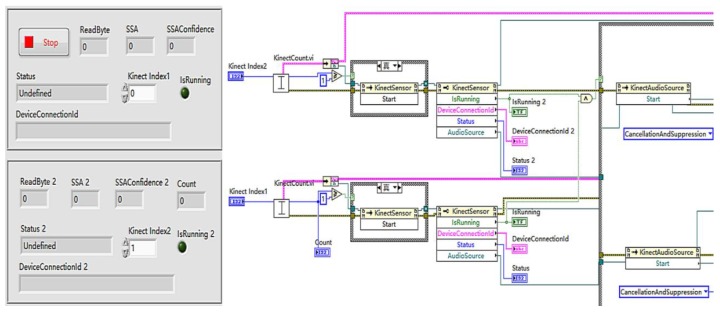
LabVIEW panel (**left**) and block diagram (**right**).

Based on the SSL method, one Kinect linear direction estimation with three microphones can determine right (θ > 0°) or left (θ < 0°) and two Kinect estimations with six microphones can synchronously localise four quadrants (Figure 4). Only Kinect direction estimations were chosen for SSL tests because its distance estimations were unsatisfactory due to their large error. Kinect microphones sensitivity and their 16 kHz sampling are important factors that can lead to delay estimation errors [28]. Time series of two Kinect sensors were consistent because of their connection to the same computer with the same measuring time. The SSL method was embedded in this online monitoring system by calling Kinects SDK in one whole loop to realise the real-time localisation function. The SSL measurements of two Kinects were LabVIEW controlled to start KinectSensor and KinectAudioSource together to invoke nodes (Kinect SDK) for synchronous acquisition at 1 s intervals (Figure 5).

The accuracy of sound source localisation is computed using:

(10) $$\;\mathrm{accuracy}=\frac{\mathrm{correct}\;\mathrm{number}\;}{\mathrm{correct}\;\mathrm{number}+\mathrm{incorrect}\;\mathrm{number}}\times 100\%$$

where accuracy is the ratio of successful localisations (SSL area (I–IV) in accordance with visual assessment of the existing laying hen area (I–IV) at 1 s intervals), correct number is the quantity of successful localisations, and incorrect number is the quantity of failing localisations (SSL area (I–IV) is inconsistent with visual assessment of the existing laying hen area (I–IV) at 1 s intervals).

### 2.4. SSL Data Processing and Storage

SSL output data were automatically pre-processed and analysed based on the LabVIEW NET module. Digital images were manually checked to evaluate accuracy of sound source localisation. Additionally, null values and invalid values were filtered through LabVIEW software automatically. Null values mean running errors of LabVIEW SSL measurements or no sound production meant the program returned null. Kinect SDK provides a confidence coefficient parameter that allows us to estimate SSL method accuracy. The threshold value of the confidence coefficient was set at 0.5 to achieve highly credible data. Also, sound source angles outside −30° to +30° were eliminated because they were beyond our scope of interest. Ineffective angles or low confidence coefficients can cause invalid SSLs, which were detected and eliminated by using a LabVIEW conditional judgement algorithm. All SSL output data were screened to determine whether they were within effective angles (−30° to +30°) and confidence coefficients (>0.5) [29]. The remaining data stored in a MySQL database were manually checked to judge whether they were in accordance with the visual assessment (successful localisation) or not (failing localisation). Besides, only laboratory tests utilised Grubbs gross error processing [30].

## 3. Results

### 3.1. Laboratory Tests

**Figure 6:**
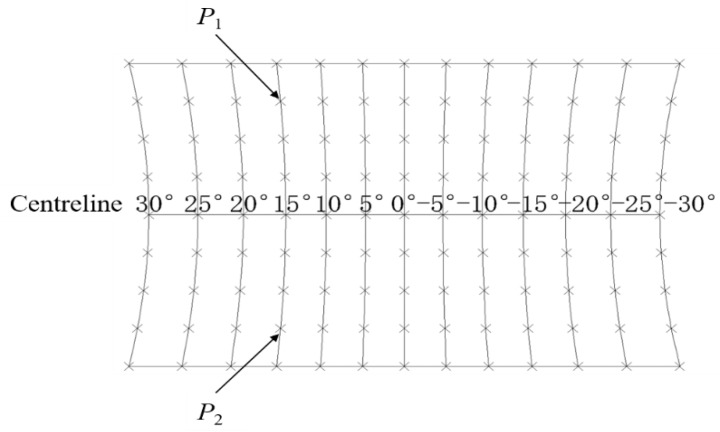
Reference values of sound source angle for a single Kinect positioned on the centreline at 0°. *P*_1_, a random sound source point; *P*_2_, a symmetry point along the centreline that has the same $r_{2}$ and θ.

In laboratory conditions, the accuracy of this system was measured as compared to previous work. Figure 6 shows the reference values of floor reference points that were regarded as an accurate way to measure errors of different angles. The centreline is the medial axis of the captured image. Different angles are distributed unevenly along the centreline. The same sound source angle θ of the Kinect at different positions is nonlinear. Table 1 presents the errors of different angle measurements between the SSL method and referenced angles. On average, the absolute error of the Kinect was lower than 2°.

Then, 1068 of the 3272 samples generated during the single Kinect test were found to be valid SSLs, and 1087 of the 3272 samples were non-null values, while 19 samples were invalid SSLs due to ineffective angles or confidence coefficients (≤0.5) (Table 2). Most null values appeared due to no sound production and the LabVIEW program returned null SSL measurements.

Limited by linear microphone arrays, a single Kinect cannot unambiguously determine source location, since SSL results can get two points with the same $r_{2}$ and θ (for instance, point *P*_1_ and *P*_2_ in Figure 6). For further analysis, two orthogonally positioned Kinects were used to realise 2D grid localisation. The accuracies of SSL of two Kinects in the laboratory experiments as determined by signal processing and analysis algorithm are presented in Table 3. The mobile phone was placed in different locations of four areas as 9 × 7 reference points and 12 points in each area were evaluated (Figure 2). Out of the 261 measurements of position in the laboratory experiments, 195 were correctly identified, a 74.7% accuracy. Out of the 72 total Area I events, the algorithm correctly recognised 66 events, a 91.7% accuracy. In addition, 3 of 21 events were incorrectly recognised in Area III. The accuracies of Area II and Area IV were relatively lower than those of other areas. The direction of the mobile phone speaker in these two areas might be closer to the centreline, around the 0° angle, which can lead to misjudgment by the Kinect low sampling rate microphones. This is probably because there is a substantial effect on angle measurement around the division between two adjacent areas. The distances between different reference points are 245 mmL and 237 mmW and the size of mobile phone is 153.6 mmL × 75.2 mmW. However, the direction of the mobile phone speaker might be an influencing factor that we did not pay much attention to.

The accuracies of SSL in one chicken test are presented in Table 4. A hen’s chirping sounds were recorded over one hour, and 159 valid SSLs were extracted via this online monitoring system. In Area I, the algorithm correctly localised 44 sound sources, and 12 were incorrectly located as coming from other areas, yielding a localisation accuracy of 78.6%. In Area II, the algorithm correctly localised 42 out of 61 and incorrectly localised 19, yielding a 68.9% localisation accuracy. Similarly, localisation accuracies for Areas III and IV were 74.1% and 73.3%, respectively. The overall localisation accuracy in all areas, on average, was 73.6%.

### 3.2. Small Group Tests

**Figure 7:**
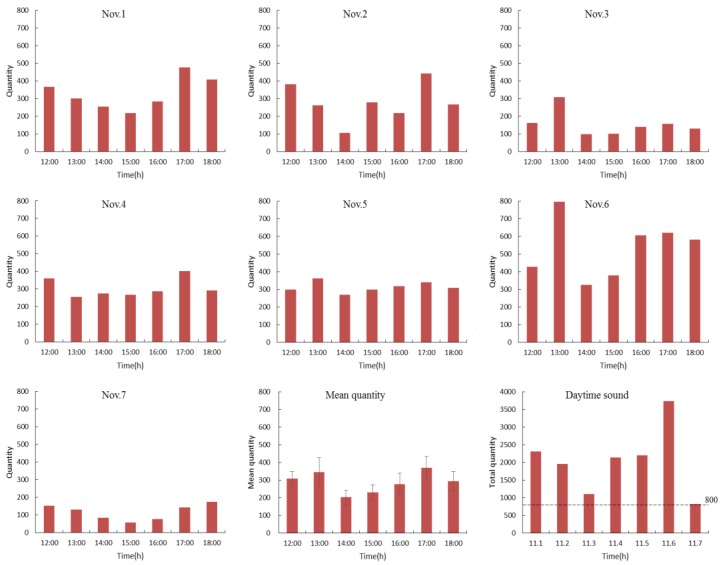
Examples of the hourly sounds of laying chickens.

**Figure 8:**
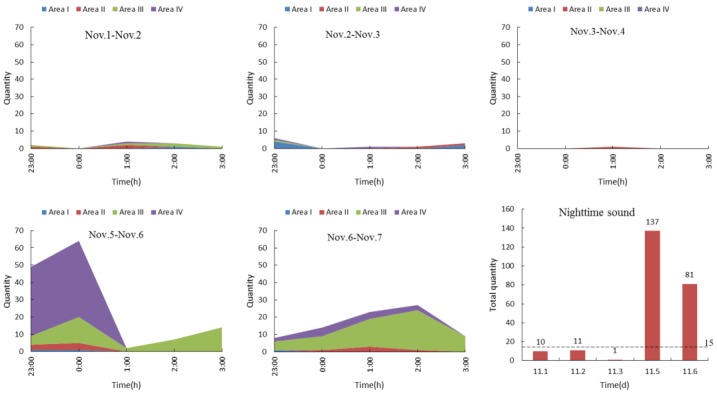
Area distribution of poultry vocalisations at night.

In the application of the SSL method to small poultry group tests, the number of hourly vocalisations (from noon to 6:00 p.m.) of the flock in the horizontal plane and the overall mean (mean ± SE) are presented in Figure 7 (data from 1–7 November were selected). The SSL method can count whether there was sound production and valid SSLs at 1 s intervals through data pre-processing of Section 2.4 or not. The number counted in an hour was the number of hourly vocalisations. It can be observed that the number of vocalisations during feeding time was higher than at any other time. The flock produced more vocalisations during feeding time, which derived from their demand or calling for food. The quantity of sounds was above 600 times (40 times per chicken) during feeding time on an average day. There is so much difference in amount of vocalisations between the days because different feeding time (normal or abnormal) as well as external stimulus might cause different vocalisation in laying hens. For example, more sounds arose on 6 November. This was the result of an egg collection causing disruption to normal feeding (1:00 p.m.) and the ensuing stress response from the flock. Lack of food resulted in more sounds arising between 4:00 p.m. and 6:00 p.m. on 6 November. The number of hourly vocalisations and area distributions (from 11:00 p.m. to 3:00 a.m.) of the flock are presented in Figure 8. The SSL method can not only count whether there was sound production and valid SSLs, but also locate to the exact sound source areas for quantitative sub area counting statistics. A high number of vocalisations arose in the daytime (noon to 6:00 p.m.), (more than 800 times or 53 times per chicken), and a minority arose during the night (11:00 p.m. to 3:00 a.m.), that is, normally fewer than 15 times (1 time per chicken) (Figure 7 and Figure 8). From noon to 6:00 p.m., laying hens might rest, socialise, eat, drink, and express their natural behaviour and the higher number of vocalisations tend to occur erratically during this period [31]. Generally, the number of vocalisations during the daytime was more random than that during the night.

Figure 8 shows the area distributions of poultry vocalisations at night (from 11:00 p.m. to 3:00 a.m.). In normal conditions, vocalisations produced by hy-line browns can hardly be heard at night, yet abnormal environmental conditions or failing management practices can cause stress to the flock, resulting in the production of more abnormal sounds. Due to late and inadequate feeding, animals were in starvation status on the nights of 5 and 6 November, which lead to increased vocalisation distributions in the feeding and drinking areas (the 4th sub chart in Figure 8). By analysing the number of poultry vocalisations and their area distributions via SSL methods, we can identify abnormal sounds (e.g., if the number of the flock’s vocalisations are obviously higher than its normal quantity at night) to find feeding problems.

**Figure 9:**
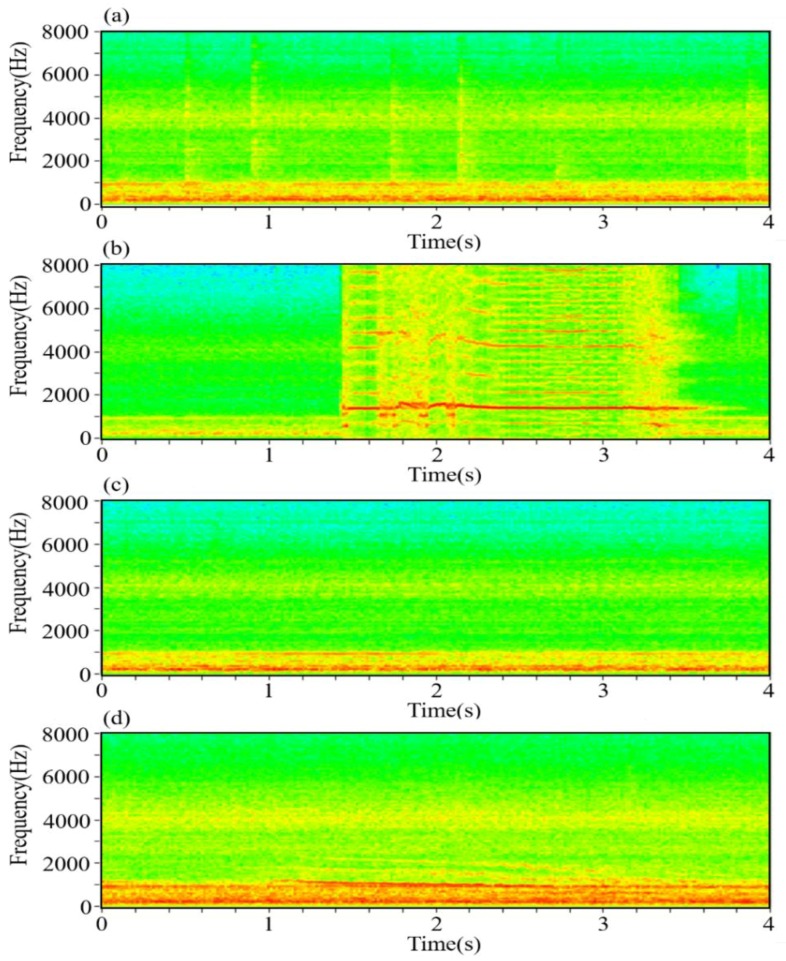
Spectrograms depicting (**a**) pecking sound for water, (**b**) cock crow sound, (**c**) mechanical sound and (**d**) chicken grunt sound.

Figure 9 presents the typical night sound spectrograms. All types of night sound were classified by human subjective hearing as well as visual inspection of spectrograms. There were four main types of sound: the pecking sound for water, cock crow sound, mechanical sound and chicken grunt sound, which were easily discriminated by playing back audio recordings. Pecking sounds for water were rarely produced except during starvation events and expressed a wide frequency range (1–8 kHz) and a short-time characteristic (Figure 9a). Cock crows were produced according to the circadian clock [32] and expressed a wide frequency range (0–8 kHz) at stable formant frequency of approximately 2 s in duration (Figure 9b). Mechanical sounds produced by fans were considered noise and were present throughout the night with irregular and random signals between 0 and 1 kHz (Figure 9c) [33]. Occasionally, chicken grunt sounds could be heard and picked up by the surveillance system but were almost impossible to capture during the daytime. They expressed a small frequency range (1–2 kHz) as well as a faint formant of 2 s duration (Figure 9d). The majority of the sound that arose in Areas III and IV relative to other areas can be accounted for by starvation or urging for food (e.g., hanging around the trough and waterline) on the nights of Nov. 5 (Areas I and II, 9 times; Areas III and IV, 127 times) and Nov. 6 (Areas I and II, 6 times; Areas III and IV, 75 times). This is because the majority of the sound that arose in areas III and IV on the nights of Nov. 5 and 6 was pecking sound for water that was discriminated by human subjective evaluation (Figure 8 and Figure 9a). These actual sound source areas were detected by the SSL method and the results were also inspected by human subjective hearing as well as inspection of spectrograms in the LabVIEW sound and vibration module.

## 4. Discussion

Due to computational efficiency as well as robustness against mismatches of signal models and microphone errors, TDOA-based source localisation approaches are perhaps the most popular. Absolute horizontal errors of the SSL method were lower than 2°, and the relative error was lower than 25.0%, within the angle measurement range of −30° to +30°. The SRP-PHAT algorithm has shown less than 4° average direction errors (horizontal and vertical) with a sound emitter played by a standard PC speaker at a distance of 1.0–3.6 m [24]. The played signal was white Gaussian noise at a 44.1 kHz sampling rate. Horizontal errors were lower than 2°, while the vertical errors were lower than 4°. The SSL method is superior to the vertical measurement of reference method and is similar to the horizontal measurement. In addition, the PHAT algorithm has shown a mean error of less than 6° at different frequencies (0.1–5 kHz) of sinusoidal Pings-Pause sound for microphone pairs 1 and 2 of a single Kinect computed at a distance of 1 m [34]. The inaccuracy was large on either side towards 0° and 180° (90° facing the Kinect), which is different to what was observed in this paper, that the error close to 0° (or facing the Kinect) tends to be larger than that facing away from the centre [34,35]. This is because the reference algorithms are based on a planar-wave beam-forming model, which is more sensitive to delay estimation errors from the sides of the linear microphone array. Another beam-forming technology showed less than 4° average angle errors with a sound box at different environmental noise levels. The range from −60° to +60° showed excellent performance at a distance of 2 m with continuous sound [35]. In short, this SSL method can accurately point to the source’s direction rather than precisely identifying its position.

With an accurate source direction, it is easy to detect the sub area in which laying hens produce sound. The system can detect abnormal status of laying hens by automatically tracking and recording the flock’s number of vocalisations as well as location. For example, when lacking food, the poultry drank more water, which is different from the standard production performance suggested by Hy-Line International. The standard feed intake and water consumption are 112–116 g/bird/day and 168–232 mL/bird/day, respectively [31]. Normally, water consumption of birds is twice the amount of their feed intake. However, in abnormal feeding status or under feed restriction, water consumption is irregular [36]. Sounds of pecking for water from birds in abnormal status might be a kind of redirected behaviour when they cannot get access to food. Additionally, these vocalisations produced by pecking water nipples might be a kind of stereotyped behaviour [37]. By analysing the number of vocalisations and their area distributions via SSL, we can seek out abnormal sounds (e.g., if the flock’s number of vocalisations is higher than the normal night-time level) and find feeding problems. A real-time monitoring algorithm based on analysing image distribution indices of broilers is used in commercial chicken houses, and some of the possible applications are detection of feeding and drinking problems, detection of malfunctions in heating or ventilation and monitoring of vaccination effects [38]. In general, the SSL method can be used for the automatic detection of problems with feeding and drinking, which has not yet been reported for laying hen populations.

In addition, the relatively lower localisation accuracy in one-chicken tests compared with laboratory tests was caused by the animal’s activities (especially fast-moving activities), as well as ambient noise. Kinect SDK provides an audio pre-processing function including echo cancellation, automatic gain control, etc. It can help us to cope with sound wave reflection. Additionally, an improved algorithm would reduce interval time to cope with problems of chickens’ fast movement in future tests. In general, the accuracy of this SSL algorithm, based on a couple of Kinects with six microphones, was above 70.0%. A reference method has an accuracy of 84.2% (16 out of 19 pig cough attacks) with eight microphones, which is more than the suggested six microphones of the SSL method while the least required four microphones [15,39]. However, the fewer the number of microphones, the lower the SSL accuracy might be. Furthermore, the next step is to verify the acceptable accuracy of SSL at various distances and to determine 3D azimuth for application in commercial houses.

Our results support the notion that poultry make little sound at night [31,32]. It was observed in this paper that a large number of laying hens’ vocalisations arose in the daytime, whereas a small number of vocalisations normally occurred at night. In normal sleep time, birds barely express vocal behaviour, except for nocturnal birds, and a minority of bird species are nocturnal birds [40,41]. In contrast, a flock of laying hens may produce many vocalisations during their feeding time, with a number of vocalisations above 600 (or 40 times per chicken) on an average day. However, the frequency of laying hens’ vocalisations during feeding time has not been reported. The researchers therefore propose the development of a real-time sound processing technology to accurately and continuously detect the feeding behaviours of broiler chickens, including feeding rate (g/min), feed intake (g) and meal duration (min) [12,13,14]. Certainly, SSL technology can be combined to assess animal health and welfare by automatically and continuously monitoring their feeding behaviour.

## 5. Conclusions

A monitoring system using microphone arrays of Kinects was developed to automatically recognise bird distributions via sound source localisation techniques. The SSL results from these Kinects had an accuracy of 74.7% and 73.6% in laboratory tests and small group tests. The flock produced an average of 40 sounds per chicken during feeding time. Particularly, the flock made significantly more sounds while undergoing stress or suffering from starvation. The system can successfully monitor the abnormal night vocalisations of poultry by analysing sound distribution areas. Additionally, it was found that the poultry flock stayed silent during their sleep time. In terms of area distribution of the poultry at night, abnormal practice management was easily discovered by using this monitoring system. Future work should reduce noise disturbance in order to better capture and quantify the flock’s SSL, and thus improve the associated implications for animal welfare and facility design adequacy. In addition, a large-scale flock test and abnormal events such as the malfunction of machines or imposition of group stress will be artificially designed to validate this system.

## Figures and Tables

**Table 1:** Errors of different angle measurements for a single Kinect in the laboratory experiment.

Angle measurement	−28°	−22°	−15°	−8°	0°	8°	15°	22°	28°
Absolute error (°)	1	2	2	2	0	2	2	1	0

**Table 2:** SSL testing information at 1 s intervals.

Day	Time (h)	Number of SSLs	Number of Non-Null SSLs	Number of Valid SSLs	Test Type	Remarks
28 October	2	1550	458	450	Laboratory	Single Kinect test
29 October	2	1722	629	618		
Total	4	3272	1087	1068		
30 October	2	751	266	261	Laboratory	Double Kinects test
31 October	1	369	164	159	One chicken	
Total	3	1120	430	354		
1 November	10	23,149	2366	2314	Small group	Continuous monitoring
2 November	14	31,459	2038	1989		
3 November	14	32,099	1123	1106		
4 November	12	23,959	2167	2139		22:00–24:00 data lost
5 November	9	15,540	2276	2249		0:00–4:00 data lost
6 November	14	23,903	3975	3897		Continuous monitoring
7 November	14	24,910	992	976		
Total	87	175,019	14,937	14,670		

Note: Valid SSLs mean effective angle within ±30°, confidence coefficient >0.5 and Grubbs gross error processing.

**Table 3:** Accuracies of sound source localisation of double Kinects in laboratory experiments.

Source Position	Number of Correct SSLs	Number of Incorrect SSLs	Total Number	Accuracy (%)
Area I	66	6	72	91.7
Area II	52	34	86	60.5
Area III	18	3	21	85.7
Area IV	59	23	82	72.0
Total	195	66	261	74.7

Note: Area partitions according to Figure 1. Correct and incorrect SSLs mean effective angle (−30° to +30°), confidence coefficient >0.5.

**Table 4:** Accuracies of SSL with two Kinects in one-chicken tests.

Source Position	Number of Correct SSLs	Number of Incorrect SSLs	Total Number	Accuracy (%)
Area I	44	12	56	78.6
Area II	42	19	61	68.9
Area III	20	7	27	74.1
Area IV	11	4	15	73.3
Total	117	42	159	73.6

Note: Area partitions according to Figure 1. Correct and incorrect SSLs mean effective angle (−30° to +30°) and confidence coefficient >0.5.

## References

[1] Manteuffel G., Puppe B., Schön P.C. (2004). Vocalization of farm animals as a measure of welfare. Appl. Anim. Behav. Sci..

[2] Exadaktylos V., Silva M., Aerts J.M., Taylor C.J., Berckmans D. (2008). Real-time recognition of sick pig cough sounds. Comput. Electron. Agric..

[3] Ferrari S., Piccinini R., Silva M., Exadaktylos V., Berckmans D., Guarino M. (2010). Cough sound description in relation to respiratory diseases in dairy calves. Prev. Vet. Med..

[4] Steen K.A., Therkildsen O.R., Karstoft H., Green O.A. (2012). Vocal-Based Analytical Method for Goose Behaviour Recognition. Sensors.

[5] Van Hirtum A., Berckmans D. (2003). Fuzzy approach for improved recognition of citric acid induced piglet coughing from continuous registration. J. Sound Vib..

[6] Ferrari S., Silva M., Guarino M., Aerts J.M., Berckmans D. (2008). Cough sound analysis to identify respiratory infection in pigs. Comput. Electron. Agric..

[7] Guarino M., Jans P., Costa A., Aerts J.M., Berckmans D. (2008). Field test of algorithm for automatic cough detection in pig houses. Comput. Electron. Agric..

[8] Silva M., Exadaktylos V., Ferrari S., Guarino M., Aerts J.M., Berckmans D. (2009). The influence of respiratory disease on the energy envelope dynamics of pig cough sounds. Comput. Electron. Agric..

[9] Zimmerman P.H., Koene P. (1998). The effect of frustrative nonreward on vocalisations and behaviour in the laying hen, Gallus gallus domesticus. Behav. Process..

[10] Evans C.S., Evans L. (1999). Chicken food calls are functionally referential. Anim. Behav..

[11] Wauters A.M., Richard-Yris M.A. (2002). Mutual influence of the maternal hen’s food calling and feeding behavior on the behavior of her chicks. Dev. Psychobiol..

[12] Aydin A., Bahr C., Viazzi S., Exadaktylos V., Buyse J., Berckmans D. (2014). A novel method to automatically measure the feed intake of broiler chickens by sound technology. Comput. Electron. Agric..

[13] Aydin A., Bahr C., Berckmans D. (2015). A real-time monitoring tool to automatically measure the feed intakes of multiple broiler chickens by sound analysis. Comput. Electron. Agric..

[14] Aydin A., Berckmans D. (2016). Using sound technology to automatically detect the short-term feeding behaviours of broiler chickens. Comput. Electron. Agric..

[15] Silva M., Ferrari S., Costa A., Aerts J.M., Guarino M., Berckmans D. (2008). Cough localization for the detection of respiratory diseases in pig houses. Comput. Electron. Agric..

[16] Yu L.G., Teng G.H., Li B.M., Lao F.D., Cao Y.F. (2013). Classification Methods of Vocalization for Laying Hens in Perch System. Trans. Chin. Soc. Agric. Mach..

[17] Cao Y.F., Chen H.Q., Teng G.H., Zhao S.M., Li Q.W. (2015). Detection of Laying Hens Vocalization Based on Power Spectral Density. Trans. Chin. Soc. Agric. Mach..

[18] Cai W.P., Wang S.K., Wu Z.Y. (2010). Accelerated steered response power method for sound source localization using orthogonal linear array. Appl. Acoust..

[19] Bower J.L., Clark C.W. (2005). A Field Test of the Accuracy of a Passive Acoustic Location System. Bioacoustics.

[20] Mennill D.J., Battiston M., Wilson D.R., Foote J.R., Doucet S.M. (2012). Field test of an affordable, portable, wireless microphone array for spatial monitoring of animal ecology and behaviour. Methods Ecol. Evol..

[21] Mennill D.J., Vehrencamp S.L. (2008). Context-Dependent Functions of Avian Duets Revealed by Microphone-Array Recordings and Multispeaker Playback. Curr. Biol..

[22] Blumstein D.T., Mennill D.J., Clemins P., Girod L., Yao K., Patricelli G., Deppe J.L., Krakauer A.H., Clark C., Cortopassi K.A. (2011). Acoustic monitoring in terrestrial environments using microphone arrays: Applications, technological considerations and prospectus. J. Appl. Ecol..

[23] Jana A. (2012). Kinect for Windows SDK Programming Guide.

[24] Seewald L.A., Gonzaga L., Veronez M.R., Minotto V.P., Jung C.R. (2014). Combining SRP-PHAT and two Kinects for 3D Sound Source Localization. Expert Syst. Appl..

[25] Kunin V., Turqueti M., Saniie J., Oruklu E. (2011). Direction of Arrival Estimation and Localization Using Acoustic Sensor Arrays. J. Sens. Technol..

[26] Jin Y., Yang R.Z. (2007). Research Status and Prospect of the Acoustic Localization Techniques. Audio Eng..

[27] Benesty J., Chen J., Huang Y. (2008). Microphone Array Signal Processing.

[28] Rascon C., Fuentes G., Meza I. (2015). Lightweight multi-DOA tracking of mobile speech sources. Eurasip J. Audio Speech.

[29] Galatas G., Ferdous S., Makedon F. (2013). Multi-modal Person Localization and Emergency Detection Using The Kinect. Int. J. Adv. Res. Artif. Intell..

[30] Grubbs F.E. (1950). Sample Criteria for Testing Outlying Observations. Ann. Math. Stat..

[31] Hy-Line Parent Stock Management Guides. http://www.hyline.com/aspx/general/dynamicpage.aspx?id=255.

[32] Shimmura T., Yoshimura T. (2013). Circadian clock determines the timing of rooster crowing. Curr. Biol..

[33] Cao Y.F., Yu L.G., Teng G.H., Zhao S.M., Liu X.M. (2014). Feature extraction and classification of laying hens’ vocalization and mechanical noise. Trans. Chin. Soc. Agric. Eng..

[34] Reddy V.R., Deshpande P., Dasgupta R. Robotics Audition using Kinect. Proceedings of the 6th International Conference on Automation, Robotics and Applications (ICARA).

[35] Wang S., Yang P., Sun H. Design and Implementation of Auditory System for Mobile Robot Based on Kinect Sensor. Proceedings of the 12th World Congress on Intelligent Control and Automation (WCICA).

[36] Tiecheng L. (2018). The importance and method of feed restriction in laying hens’ growing period. Mod. Anim. Husb. Sci. Technol..

[37] Kuhne F., Sauerbrey A.F.C., Adler S. (2013). The discrimination-learning task determines the kind of frustration-related behaviours in laying hens (Gallus gallus domesticus). Appl. Anim. Behav. Sci..

[38] Kashiha M., Pluk A., Bahr C., Vranken E., Berckmans D. (2013). Development of an early warning system for a broiler house using computer vision. Biosyst. Eng..

[39] Spiesberger J.L. (2001). Hyperbolic location errors due to insufficient numbers of receivers. J. Acoust. Soc. Am..

[40] Digby A., Towsey M., Bell B.D., Teal P.D. (2014). Temporal and environmental influences on the vocal behaviour of a nocturnal bird. J. Avian Biol..

[41] Perrault K., Lobert L.M., Ehnes M., Foote J.R. (2014). Nocturnal singing in a temperate bird community. J. Ornithol..

